# Narcotic requirements after shoulder arthroplasty are low using a multimodal approach to pain

**DOI:** 10.1016/j.jseint.2021.02.005

**Published:** 2021-04-06

**Authors:** Paul M. Sethi, Nikhil K. Mandava, Nicole Liddy, Patrick J. Denard, Georges Haidamous, Charles D. Reimers

**Affiliations:** a Orthopedic and Neurosurgery Specilaists Foundation, Greenwich, CT, USA; b Southern Oregon Orthopaedics, Medford, OR, USA

**Keywords:** Shoulder, Surgery, Arthroplasty, Narcotic, Reduction, Liposomal, Bupivacaine, Opioids

## Abstract

**Background:**

Recent “multimodal” approaches to pain, although understudied, have shown promise in reducing reliance on narcotics in shoulder arthroplasty (SA). Many surgeons report being unsure of how many narcotic pills to prescribe after the surgery. As result, patients are prescribed upwards of 60 oxycodone 5-mg pills for a 6-to-12-week treatment period despite studies showing postoperative pain can be managed without any medication at all.

**Purpose:**

The purpose of this multicenter study was to prospectively determine the number of opiate pills required after SA to develop generalizable, evidence-based prescription guidelines for surgeons. We hypothesized that opioid prescription would be low using a multimodal approach to pain management.

**Methods:**

The study enrolled 63 patients undergoing SA. Subjects received either an interscalene nerve block with liposomal bupivacaine, standard bupivacaine, or a local infiltration standard bupivacaine field block based on preference. All subjects were provided with postoperative “Pain Journals” to document their daily pain on a Numerical Rating Scale and daily opioid consumption during the 14-day postoperative period.

**Results:**

Overall, patients consumed an average of 8.6 oxycodone 5-mg pills (64.5 morphine milligram equivalents) after SA. Seventy-nine percent of patients required 15 or fewer oxycodone 5-mg pills, and 27% successfully managed their postoperative pain with zero opioids. Average pain remained low for patients in all groups.

**Conclusion:**

With a multimodal approach, most patients undergoing SA can manage postoperative pain with 15 or fewer oxycodone 5-mg tablets, or 112.5 morphine milligram equivalents. The addition of a liposomal bupivacaine interscalene nerve block may further reduce the consumption of postoperative narcotics compared with a standard interscalene nerve block. This study provides evidence that may be used for surgeon guidelines in the effort to reduce opioid prescriptions after SA.

Nearly 100,000 combined total and reverse shoulder arthroplasties (SAs) are performed annually in the United States, a number expected to double over the next 5 years.^7^ At the same time, outpatient total SA (TSA) is becoming more commonplace. Crucial to this progression is adequate postoperative pain management.^2^ Prescription narcotics have been a historic mainstay of pain management in this regard. However, opioid prescriptions have risen exponentially in recent decades along with associated deaths.^12^ Recently, the movement to administer a “multimodal” array of analgesics has shown promise in reducing institutional and individual reliance on opiate medication but remains understudied.^2^^,^^4^^,^^6^^,^^8^^,^^24^

The scarcity of objective prescription guidelines after SA forces orthopedic surgeons to make educated guesses regarding patient pain control. Nearly 15% of opioid-naïve patients treated with opioids after shoulder surgery will continue to consume narcotics beyond 180 days, and more than 50% of patients receiving 90 days of continued opioid therapy are chronic users after 5 years.^13^^,^^14^ Consensus-based panels recommend 50 or more oxycodone 5-mg pills for pain management after shoulder surgery.^4^^,^^26^ Accordingly, shoulder surgeons still prescribe an average of 432.5 morphine milligram equivalent (MME) (58 oxycodone 5-mg pills) and report treating postoperative pain over a 6- to 12-week period, directly contrasting quantified risks for opioid dependence outlined by the Center for Disease Control and Prevention.^5^^,^^22^^,^^25^ At the same time, Leas at al.^12^ suggest that postoperative pain after SA can be managed without any narcotic medication at all. There is a clear inconsistency between institutional expert panel opinions and risk of opiate dependence that may further perpetuate a culture of opioid overprescription.^15^^,^^17^^,^^26^

The purpose of this multicenter study was to prospectively determine the number of opiate pills required after SA to develop generalizable, evidence-based prescription guidelines for surgeons. We hypothesized that opioid prescription would be low using a multimodal approach to pain management.

## Materials and methods

A prospective evaluation was performed of patients who underwent SA between November 2018 and March 2019 at 2 centers: Orthopaedic Neurosurgery and Specialists (Greenwich, CT, USA) or Southern Oregon Orthopaedics (Medford, OR, USA). Institutional review board approval was obtained before commencing the study (Greenwich Hospital IRB #2018016). Eligible subjects were those undergoing primary anatomic TSA or reverse SA, minimum of 18 years of age, willingness to fill out the “pain journal,” ability to understand the informed-consent process and to document informed consent before completion of any study-related procedure, and ability to read, comprehend, and complete subject-reported outcome measures in English.

Subjects were excluded for documented history of drug or alcohol abuse, use of narcotic painkillers for greater than 3 months before surgery, neurologic deficit or disability involving the surgical extremity, known allergy to amide anesthetics, known allergy or intolerance to hydrocodone or oxycodone, currently enrolled or planning to enroll in another clinical trial during this study that would affect the outcome of this study, and/or history of a cognitive or mental health status that would interfere with study participation.

Ninety-seven SAs were performed during the study period and assessed for study eligibility. These procedures were completed by 3 fellowship-trained surgeons. Indications for surgery included degenerative joint disease, rotator cuff arthropathy, and proximal humeral fracture sequelae. Fourteen patients refused to participate, 8 were excluded for preoperative narcotic usage, and 3 were excluded for revision surgery. Thus, 72 patients were enrolled in the study. Nine patients did not turn in their pain journals, leaving 63 patients (87.5%) with complete follow-up.

### Pain management

**Figure 1:**
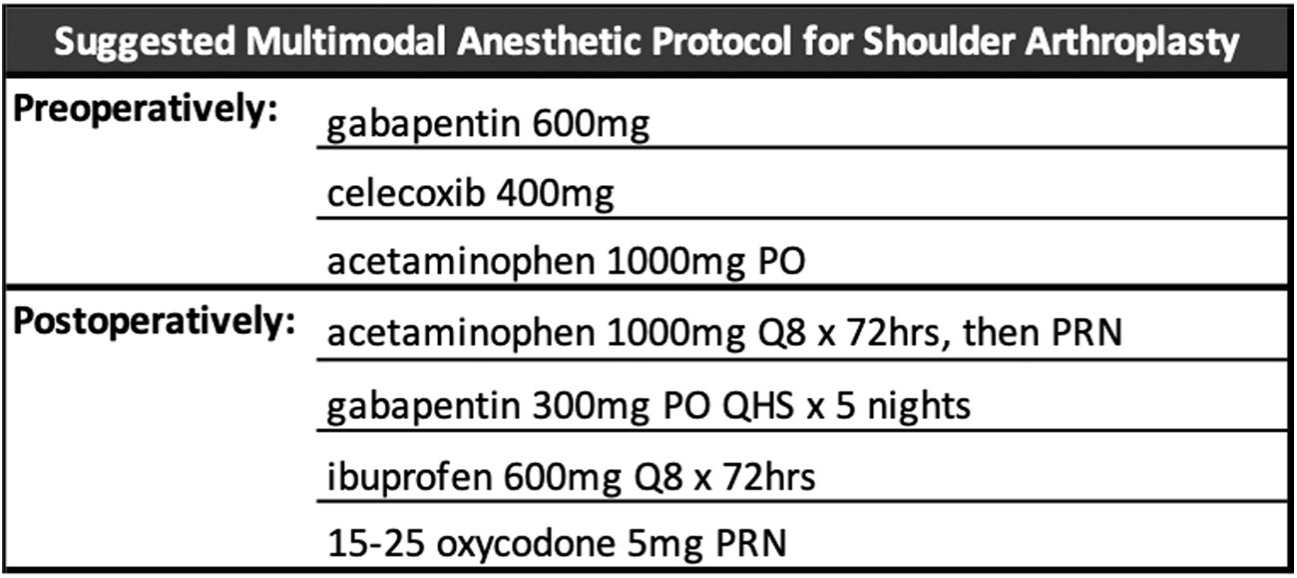
Study anesthetic protocol using liposomal bupivacaine or bupivacaine interscalene nerve block for rotator cuff repair. It includes 2 oral (PO) medications to be administered preoperatively, an ultrasound-guided interscalene block to be administered by the anesthesiologist, and 3 PO medications to be administered postoperatively. *Q4*, every 4 hours; *Q8*, every 8 hours; *QHS*, before bed.

All patients received the same preoperative and postoperative oral multimodal protocol for pain management (Fig. 1). Preoperatively, subjects received 1000 mg of acetaminophen per os, 400 mg of celecoxib, 600 mg of gabapentin per os within 1 hour before surgery. Postoperatively, patients were instructed to take 1000 mg of acetaminophen and 600 mg ibuprofen scheduled for 72 hours and 300 mg of gabapentin for 3 nights and were prescribed 5 mg oxycodone (15-25 pills) instructed to be taken as needed for pain that was not controlled by the acetaminophen/non steroidal anti-inflammatory drug regimen. Patients were also instructed not to exceed 2 pills of oxycodone 5 mg within a 6-hour window.

Intraoperative analgesia was based on surgeon and patient preference after discussion of risk and benefits of an ultrasound-guided interscalene nerve block (ISNB). Subjects were administered either an ISNB with 25 mL of 0.5% bupivacaine with 10 mg of IV Decadron (SB), a solution containing 15 mL of 0.5% bupivacaine and 10 mL liposomal bupivacaine with 10 mg of IV Decadron (LB), or a local field block using a solution of 20 mL liposomal bupivacaine 13.3mg/ml with 20mL normal saline (FB). A total of 29 subjects were allocated to the LB condition, 24 subjects to the SB condition, and 10 to the FB condition based on patient and surgeon preferences.

### Data collection

**Figure 2:**
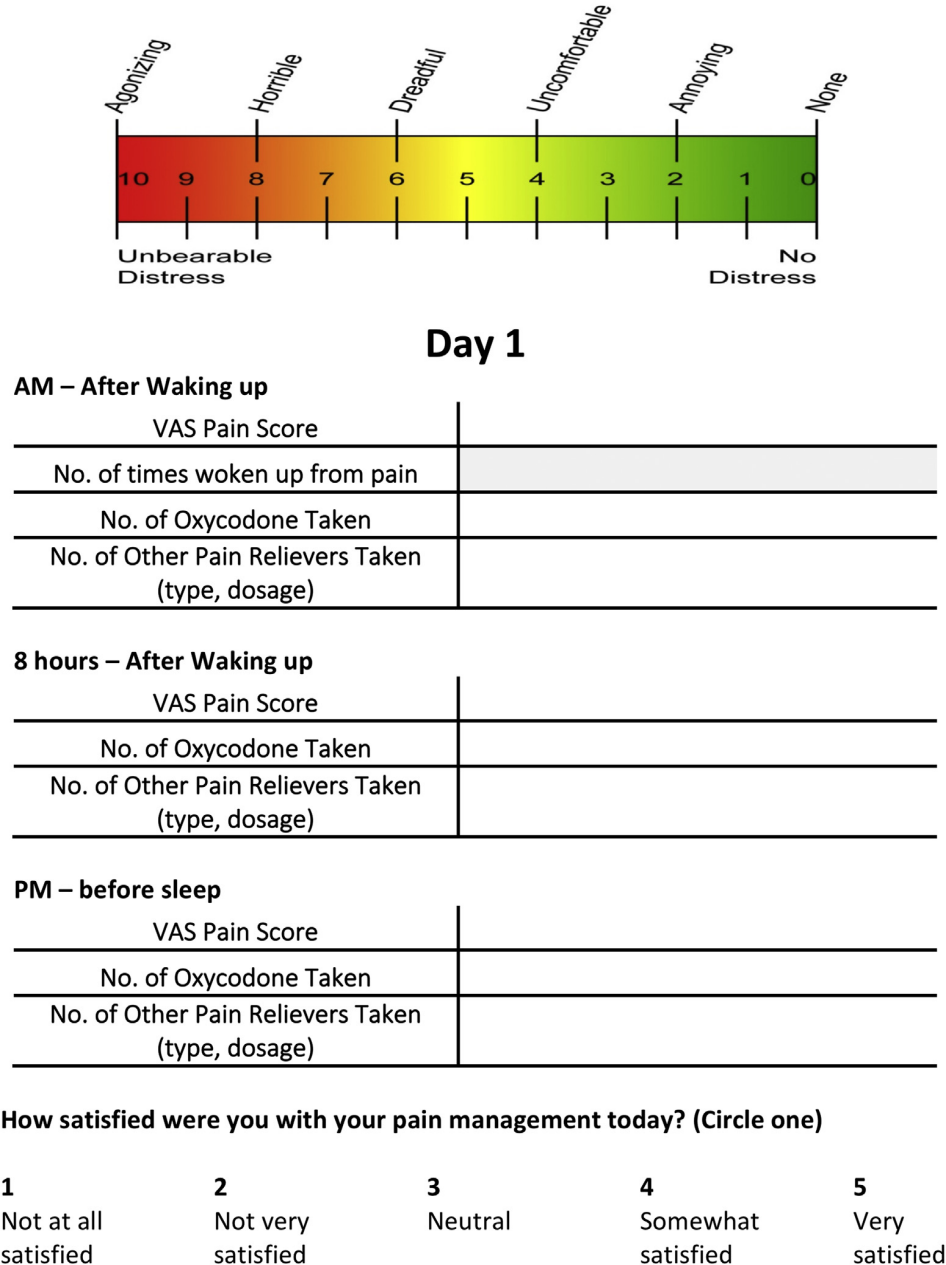
Page 2 of Shoulder Surgery Pain Journal. Subjects were instructed to complete this page at home for each postoperative day. The gradient at the *Top* of the page was provided as a reference for patients to rate their pain on a scale of 1 to 10. “VAS pain score” was more accurately described as NRS pain score during analysis.

Subjects were provided with a postoperative pain journal to be filled out on the first 5 postoperative days (PODs) and on PODs 7 and 14 as a means to collect data on pain scores and narcotic consumption (Fig. 2). At the time of enrollment, each subject was explained the correct method for recording pain scores using the provided numerical pain-rating scale and narcotic pill consumption every 8 hours concurrently with the doses of Tylenol (Johnson & Johnson, New Brunswick, NJ, USA). Numerical Rating Scale (NRS) responses were indicated on a visual scale from 0 (no distress) to 10 (agonizing pain).

Immediately after the surgery, NRS pain scores for POD 0 and any administered pain medication were recorded while the subject was fully alert in the postanesthesia care unit. Once discharged, the subject independently recorded his or her numerical pain-rating scale pain score and narcotic pill consumption every 8 hours for the remaining PODs. Subjects were called on PODs 1 and 5 to confirm adequate pain control and were reminded to complete the pain journal. Completed pain journals and the subjects’ remaining opioid medication were presented at the initial postoperative visit. Here, physicians collected the journals and counted the number of remaining narcotic pills, thus completing their involvement in the study.

### Statistical analysis

Data from each journal were organized into an Excel spreadsheet analyzed through calculations of means, standard deviations, and time-trend analysis. The NRS scores, recorded 3 times each day, were averaged to obtain a single daily score accounting for daily and nocturnal fluctuations in pain. Narcotic consumption was analyzed as a cumulative daily value in both MMEs and number of oxycodone 5-mg pills.

Despite associated biases with observational data, Chi-squared tests were used to address the statistical significance of categorical comparisons between groups. One-way analysis of variance tests were performed to compare mean pain scores and narcotic consumption among the 3 groups (LB, SB, and FB). The alpha value was set at 0.05 for significance.

## Results

The mean patient age was 69 years; of 63 patients, 30 (48%) were men and 25 (39.7%) underwent reverse SA. There were no observed differences in opioid consumption (*P* = .13) or pain (*P* = .052) between reverse SA and TSA.

Demographic data for each ISNB group are presented in Table I.

**Table I:** Demographic data of each ISNB group. The table includes the mean ages and BMI scores of each group, including the *P* value to demonstrate nonstatistical significance. Also presented are the male and female percentages per group, the percentages of smokers, and the percentages of patients with diabetes mellitus 2.

Demographics	Group			*P* value
	Exparel (LB)	Standard block (SB)	Field block (FB)	
Mean age (yr)	68.1	71.8	70.2	.09
Mean BMI score	26.2	31.6	29.4	.06
Percent female (%)	48.6	37.5	84.6	
Percent male (%)	51.4	62.5	15.4	
Percentage of smokers (%)	2.7	5	0	
Percentage of patients with diabetes mellitus 2 (%)	5.4	2.5	7.7	

*FB*, standard bupivacaine field block; *LB*, liposomal bupivacaine nerve block; *SB*, standard bupivacaine nerve block.

### Postoperative pain and narcotic usage

Across all 3 groups, patients consumed an average total of 8.6 oxycodone 5-mg pills (64.5 MMEs) after SA. Average narcotic consumption was 1.1 pills (8.22 MMEs) per day (Table II). Of 63 patients, 17 (27%) required zero opioids to manage their postoperative pain. Of 63 patients, 50 (79%) required 15 or fewer oxycodone 5-mg pills. Only 7 of 63 (11%) patients reported continuing the use of opioids at their 2-week follow-up appointment. Overall, subjects’ average daily pain rating was 1.9 NRS points (Table II). Average cumulative NRS pain, defined as the area under the curve for the 8 measured time points, was 16.5 (Table II).

**Table II:** Summary values per Postoperative Day (POD). The table presents average cumulative and average daily narcotic consumption and NRS pain ratings for each of the 8 measured PODs. These values are an average of all 3 groups observed in this study, which provides generalizable results for all patients with TSA. The “Total Avg.” column averages present the value when averaging across the entire study period.

Metric	POD 0	POD 1	POD 2	POD 3	POD 4	POD 5	POD 7	POD 14	Total average
Daily NRS	1.7	2.6	2.4	2.3	2.3	2.3	1.9	1.0	1.9
Cumulative NRS	1.7	4.3	6.7	9.1	11.4	13.6	15.5	16.5	16.5
Daily MME	6.03	20.93	11.21	8.15	6.81	6.23	3.87	1.11	8.22
Cumulative MME	6.03	26.96	38.17	46.33	53.13	59.36	63.23	64.34	64.34
Daily pills∗	0.8	2.8	1.5	1.1	0.9	0.8	0.5	0.1	1.1
Cumulative pills∗	0.8	3.6	5.1	6.2	7.1	7.9	8.4	8.6	8.6

*MME*, morphine milligram equivalent; *NRS*, Numerical Rating Scale.

∗ pills = oxycodone 5 mg pills.

### Pain and narcotic usage as per intraoperative analgesia

**Figure 3:**
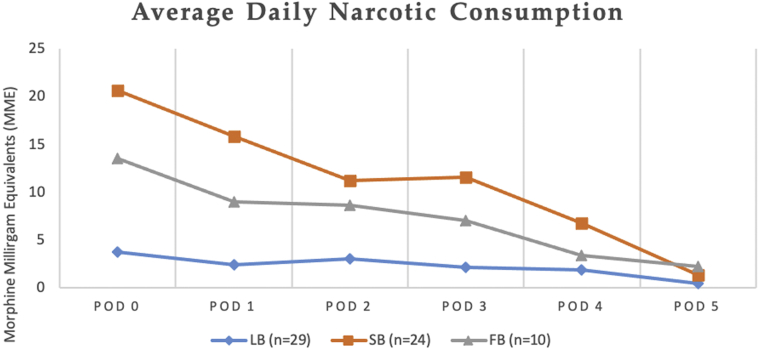
Postoperative opiate consumption (in morphine milligram equivalents) for subjects undergoing shoulder arthroplasty (SA). The chart presents the average daily NRS pain scores recorded by subjects undergoing SA with liposomal bupivacaine nerve block (LB), standard bupivacaine nerve block (SB), or standard bupivacaine field block (FB) on each postoperative day (POD).

**Figure 4:**
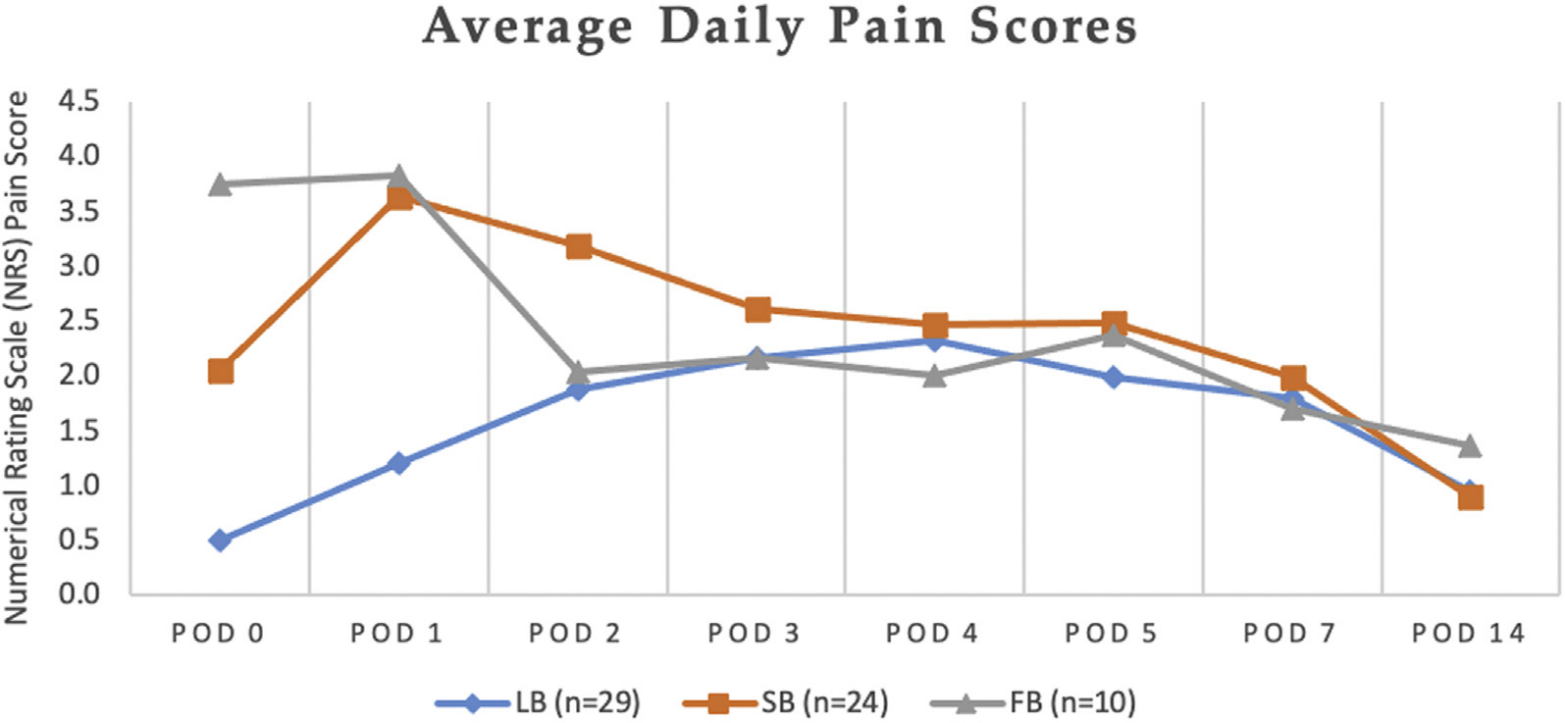
Postoperative pain measured by Numerical Rating Scale (NRS) for subjects undergoing shoulder arthroplasty (SA). The chart presents the average daily NRS pain scores recorded by subjects undergoing SA with liposomal bupivacaine nerve block (LB), standard bupivacaine nerve block (SB), or standard bupivacaine field block (FB) on each postoperative day (POD).

Figures 3 and 4 depict daily fluctuations in mean postoperative narcotic consumption and pain scores, respectively, for each group across the 8 measured time points. Subjects receiving a LB block consumed a total of 3.1 narcotic pills (20.81 MMEs) over the 14 PODs, had a cumulative NRS pain of 10.4, and an average daily NRS pain score of 1.4. Subjects receiving a SB block consumed a total of 13.6 narcotic pills (109.43 MMEs) over the 14 PODs and had a cumulative NRS pain of 19.0 and reported an average daily NRS pain score of 2.4. Subjects receiving an FB block consumed 13.2 narcotic pills (98.63 MMEs) over the 14 PODs and had cumulative NRS pain of 18.9 and an average daily NRS pain score of 2.4.

Zero narcotics were needed in 55% of LB ISNB subjects vs. 4% and 0% in the SB and FB groups, respectively (*P* < .001) (Table III). In addition, every patient in the LB group consumed 15 or fewer narcotic pills. Ninety-three percent of patients in the LB group did not consume their first narcotic pill in the first 24 hours after the surgery vs. 38% (9 of 24) in the SB group and 10% (1 of 10) in the FB group (Table III) (*P* < .001). Moreover, the LB group consumed significantly fewer narcotics (*P* < .0000001) and had less pain (*P* < .0000001) on average throughout the entire study period.

**Table III:** Pill distribution. The table presents the number of subjects that consumed a certain range of oxycodone 5-mg pills over the 14-day postoperative period, separated by block type.

Number of pills consumed by the group					
Group	0 pills	1 to 5 pills	6 to 10 pills	11 to 15 pills	15+ pills
LB	16	8	2	3	0
SB	1	4	6	3	10
FB	0	1	5	1	3

*FB*, standard bupivacaine field block; *LB*, liposomal bupivacaine nerve block; *SB*, standard bupivacaine nerve block.

## Discussion

The principal finding of this study is that nearly 80% of patients require 15 or fewer oxycodone 5-mg pills (112 MMEs) with a multimodal approach to pain after SA, regardless of intraoperative analgesia. Patients maintained this low narcotic usage while maintaining an average pain rating of less than 2 NRS points. The use of LB may further improve on this recommendation, as only 2 subjects receiving an LB block required opioids before POD 2. However, further study is needed to more accurately compare the efficacy of LB ISNB to current anesthetic techniques.

Developing evidence-based guidelines to minimize opioid prescription presents an opportunity for orthopedic surgeons to address the continued abuse of opioid medication in the United States.^3^^,^^6^^,^^19^^,^^23^^,^^25^ The Center for Disease Control and Prevention has reported increased risks of long-term opioid use after only 5 days of therapy and recommends that therapy extend no more than 7 PODs.^5^^,^^22^ The effects of smaller and shorter prescriptions are twofold, as the amount of narcotics subjects are prescribed is directly related to consumption.^6^ Furthermore, limiting excess opioids reduces their availability for recreational use and related overdoses.^11^

Based on our findings, we recommend that 15 oxycodone 5 mg pills should be used as a guideline after SA. The present study’s recommendation contrasts with multidisciplinary consensus-based panel’s recommendations. The expert panel reported by Wyles et al^26^ concluded that surgeons should prescribe 50 oxycodone 5-mg tablets (200-320 MMEs) after TSA. Median and average consumption in the present study were significantly lower than these recommendations. Martusiewicz et al^15^ provided evidence-based recommendations of 25-30 oxycodone 5-mg pills (187.5-225 MMEs) after anatomic TSA. The study by Martusiewicz et. al,^15^ however, differs from the present study in that it included subjects with preoperative narcotic consumption, a demographic factor associated with increased opioid requirements. Furthermore, low requirements in the present study support the efficacy of a voluntary opioid-free trial conducted by Leas et al.^12^ The present study suggests that at least 27% of the general population have the capacity to manage postoperative pain after TSA without opioid medication.

The low usage of narcotics in the present study is likely owing to the multimodal approach outlined in Figure 1. Multimodal analgesics including Tylenol, nonsteroidal anti-inflammatory drugs, and gabapentin have shown promise in limiting pain and reducing reliance on narcotic medication postsurgically.^2^^,^^4^^,^^6^^,^^8^^,^^24^ However, the exact combination of these components has yet to be fully elucidated. Proper education on postoperative pain expectations and narcotic use has been demonstrated to reduce postoperative pain, narcotic consumption, and risks for dependence. Although not quantified in the present study, it is likely that preoperative patient education by the surgeons influenced the low narcotic usage.

The effect of LB usage after TSA has varied results. Patel et al^18^ demonstrated a reduction in pain and opioid consumption in subjects that received an ISNB using small doses of LB when compared with saline controls. Sethi et al^21^ demonstrated that the addition of LB to multimodal anesthetic protocols significantly reduces acute perioperative pain on days 1 and 2 as well as narcotic consumption compared with standard bupivacaine in rotator cuff repair. Hannan et al^8^ showed 36-hour reductions in cumulative pain and opioid consumption on PODs 2 and 3 with LB local infiltration (LIA) compared with ropivacaine ISNB in subjects undergoing SA. Vandepitte et al^24^ and Okoroha both found LB LIA subjects had lower peak pain and enhanced satisfaction compared with standard ISNB in SA.^20^ In contrast, Abildagaard et al^1^ demonstrated higher Visual Analogue Score and narcotic consumption on POD 0, as well as an increase in cumulative narcotic requirements in SA LB LIA conditions. Namdari et al^16^ demonstrated that subjects receiving LB LIA in SA consumed more MMEs over 72 hours and had similar Visual Analogue Score compared to standard bupivacaine ISNB. Finally, Sabesan et al showed no difference in pain and narcotic consumption between LB LIA and standard bupivacaine indwelling catheter over the first 24 hours in SA. Length of stay for both groups was also similar.^20^ In the present study, the LB ISNB group all consumed fewer than 15 pills and required zero narcotics more often. They also reported less pain, although the mean pain score for any group was no higher than 2.4 at 2 weeks after the surgery. Any potential analgesic benefits of LB must first be weighed against its increased cost. Single doses of LB are 100 times more expensive than standard bupivacaine per dose.^9^ While decreased length of stay and outcome improvements can mitigate upfront costs and may present cost-saving opportunities,^10^ further study in the cost-benefit of LB ISNB vs. other anesthetic techniques is needed to better understand these nuances.

Patient outcomes using SB vs. FB have been investigated in other studies, finding similar use in average narcotic consumption per day, cumulative NRS pain score, and average daily NRS pain score. Over the 14 PODs, 13.6 and 13.2 narcotic pills were taken for SB and FB trials respectively. The average daily NRS pain score for both groups was 2.4, and the cumulative NRS pain score was 19 for the SB block group and 18.9 for the FB block group. These findings align with those of Sicard et al, who demonstrated that the use of LIA is not less effective than interscalene nerve block for pain control after TSA. Sicard et al recorded no significant difference in the average pain score for the 48-hour postoperative period between both LIA and interscalene nerve block groups. However, it was noted that in the recovery room postoperation, the LIA group consumed less opioids and had significantly less severe pain; a finding which was not noted in this study.

### Study limitations

There are several limitations to the present study. First, patients were not blinded or randomized to the various "block"-type treatments. These conditions were instead allocated based on patient and surgeon preference. Nonrandom distribution is a major source of bias that reduces the present study’s ability to determine whether one group performed better than the others regarding postoperative pain and narcotic consumption. Nonnormal distributions and high variance between groups present further limits these comparisons. However, as a level III cohort study, the generalizable observational data provided in this study can be used toward creating overarching prescription guidelines for physician use. Irrespective of the anesthesia block given or lack thereof, patients can manage postoperative pain with 15 or fewer oxycodone 5-mg tablets after the shoulder surgery. Second, subjects were informed they were taking part in a trial on postoperative pain through the recruitment process. This introduces the potential for response bias (ie, aligning their pain scores with researchers’ expectations) as well as a demand characteristic, whereby keeping an active record of their pain and narcotic consumption for the purposes of this study led subjects to subconsciously change their behavior. Third, we did not collect any data on pain or narcotic consumption between PODs 7 and 14. Minimal change was observed after day 3, yet it is possible that pain spikes were unintentionally omitted from the analysis. Finally, these findings are limited to patients not chronically taking narcotics. Patients were excluded if they took opioids within 3 months before the surgery. Therefore, the findings are not necessarily generalizable to a population with chronic opioid usage.

## Conclusion

With a multimodal approach, most patients undergoing SA can manage postoperative pain with 15 or fewer oxycodone 5-mg tablets, or 112.5 MMEs. The addition of a LB ISNB may further reduce the consumption of postoperative narcotics compared with a standard ISNB. This study provides evidence that may be used for surgeon guidelines in the effort to reduce opioid prescriptions after SA.

## Disclaimers:

*Funding:* No funding was disclosed by the author(s).

*Conflicts of interest:* Patrick J Denard is consultant for and receives royalties from Arthrex and is a paid speaker for Pacira. Paul M Sethi is a paid consultant for Arthrex and paid speaker for Pacira. The other authors, their immediate family, and any research foundation with which they are affiliated did not receive any financial payments or other benefits from any commercial entity related to the subject of this article.
